# Association of celiac disease with eosinophilic esophagitis: Nationwide register-based cohort study with sibling analyses

**DOI:** 10.1016/j.jacig.2024.100254

**Published:** 2024-04-10

**Authors:** Niki Mitselou, Amiko Uchida, Bjorn Roelstraete, Erik Melén, John J. Garber, David Katzka, Benjamin Lebwohl, Peter H.R. Green, Jonas F. Ludvigsson

**Affiliations:** a Department of Pediatrics, Faculty of Medicine and Health, Örebro University, Örebro, Sweden; b Division of Gastroenterology, Hepatology & Nutrition, University of Utah School of Medicine, Salt Lake City, Utah; c Department of Medical Epidemiology and Biostatistics, Karolinska Institutet, Stockholm, Sweden; d Department of Clinical Science and Education Södersjukhuset, Karolinska Institutet, Stockholm, Sweden; e Gastrointestinal Unit, Massachusetts General Hospital, Harvard Medical School, Boston, Mass; f Department of Medicine, Columbia University College of Physicians and Surgeons, New York, NY

**Keywords:** Celiac disease, eosinophilic esophagitis, cohort, epidemiology

## Abstract

**Background:**

Celiac disease (CeD) is associated with several immune-mediated disorders, but it is unclear whether it is associated with eosinophilic esophagitis (EoE).

**Objective:**

We sought to examine the risk of EoE in patients with biopsy-verified CeD compared with matched controls and siblings.

**Methods:**

Using nationwide population-based histopathology data, we identified 27,338 patients with CeD diagnosed in the period 2002 to 2017 in Sweden. Patients with CeD were age- and sex-matched with up to 5 reference individuals (n = 134,987) from the general population. Cox Regression was used to estimate hazard ratios (HRs) for developing biopsy-verified EoE. In a secondary analysis, we used unaffected siblings of patients with CeD as comparators to adjust for intrafamilial confounding.

**Results:**

The median age at CeD diagnosis was 27 years, and 63.3% were female patients. During a median follow-up of 8.1 years, 17 patients with CeD and 13 matched reference individuals were diagnosed with EoE. This corresponded to incidence rates of 0.08 versus 0.01 per 1000 person-years, respectively, and an adjusted HR for EoE of 6.65 (95% CI, 3.26-13.81). Compared with their siblings without CeD, patients with CeD were however at a no increased risk of EoE (HR, 1.39; 95% CI, 0.55-3.51).

**Conclusions:**

In this study, individuals with CeD were at a 6.6-fold increased risk of later EoE compared with the general population. This association might be explained by an altered health-seeking behavior or through shared genetic or early environmental factors because the excess risk disappeared in sibling analyses.

Celiac disease (CeD)^1^ and eosinophilic esophagitis (EoE)^2^ are 2 distinct immune-mediated and chronic disease entities affecting the gastrointestinal (GI) tract. CeD is characterized predominantly by an immune response triggered by dietary gluten exposure in genetically predisposed individuals, leading to small intestinal inflammation, villus atrophy, and various GI and systemic symptoms.^1^ CeD affects individuals of any age or sex, and its prevalence has been estimated to be about 1% worldwide and has increased over time in recent decades.^1^^,^^3^ However, driven by a dysregulated T_H_2 immune response, EoE is clinically characterized by esophageal dysfunction and histologically by eosinophil-predominant inflammation.^4^^,^^5^ The incidence of EoE is increasing, independently of surveillance and detection.^6^^,^^7^ A 2015 meta-analysis of 13 studies from North America, Europe, and Australia reported an overall pooled incidence rate (IR) of 3.7 per 100,000 person-years (95% CI, 1.7-6.5), although the included studies presented high heterogeneity.^8^

Recently, the growing recognition of EoE worldwide has motivated an increasing interest in the relationship between CeD and EoE, with some evidence pointing to a positive association between the 2 disorders,^9^ especially in children.^10^^,^^11^ However, a 2014 systematic review based on 30 published studies demonstrated significant publication bias in favor of case reports and short series of patients who share both conditions, showing associations between the 2 entities.^12^ All in all, the linkage of CeD with EoE remains controversial, with several case reports and cohort studies reporting varied results, whereas the quality of the available data is still only moderate, with most articles presenting methodological inconsistencies.^12^

Here, we used a validated nationwide population cohort and aimed to provide population-based risk estimates for EoE in patients with biopsy-verified CeD to better understand the relationship between these diseases.

## Methods

### Study design and population

This Swedish cohort study used prospectively collected data from national diagnostic and histopathology registers. Through the personal identity number, a unique identifier assigned to all Swedish residents at birth, we were able to link data between different national registers.^13^ In this study, we used data from the Epidemiology Strengthened by histoPathology Reports in Sweden (ESPRESSO) histopathology cohort,^14^ the Total Population Register (TPR),^15^ and the National Patient Register (NPR).^16^

The ESPRESSO cohort contains GI histopathology data from more than 6 million biopsies registered according to the Systematized Nomenclature of Medicine Clinical Terms (SNOMED-CT) and collected between 1965 and 2017 from all 28 Swedish pathology departments. During the 2002 to 2017 study period, we identified all individuals with biopsy-verified CeD by searching for topographic codes (T64-T65) and SNOMED codes related to CeD (D6218x) and small intestinal villus atrophy (M58, M5800, M58000, M58001, M58005, M58006, and M58007). Analyses were restricted to patients with CeD without a previous diagnosis of EoE. The follow-up time was from 2002 to 2017, whereas individuals who emigrated during the study period or were diagnosed with EoE before biopsy date/matching date were excluded.

The Swedish TPR is widely used for epidemiological research purposes, because it contains data on birth (including date and country of birth), sex, death, residence, and emigration.^15^ Established in 1964, the Swedish NPR includes hospital-based inpatient and, since 2001, nonprimary outpatient data. The register uses the Swedish International Classification of Diseases system to code diagnostic data, with a positive predictive value (PPV) for most chronic diseases ranging from 85% to 95%.^16^

### Reference individuals from the general population

Each patient with CeD was matched with up to 5 reference individuals who were identified through the Swedish TPR^15^ and matched for age, sex, geographic region, and calendar year of diagnosis. We included only reference individuals without previous EoE. If a reference individual developed CeD during the study period, their follow-up was censored.

### Siblings

In secondary comparisons, we used siblings as comparators to reduce the influence of shared genetics and early-life environmental exposures. Unaffected full siblings of individuals with CeD were identified through the TPR and the Multigeneration Register.

### Covariates

Through the TPR, we retrieved information on education: compulsory school (≤9 years); upper secondary school (10-12 years); and college or university (≥13 years). Similarly, data on other concomitant autoimmune disorders in both patients with CeD and reference individuals at baseline were collected from the NPR.

### Outcome

EoE was defined as having a biopsy-verified EoE diagnosis (SNOMED code M47150 in the esophagus T62). A validation study found that the PPV for this definition is 89% for EoE.^17^

### Statistical analysis

We used the Cox proportional hazards regression to estimate hazard ratios (HRs) and their 95% CIs for incident diagnosis of EoE. Follow-up time started on the date of CeD diagnosis or corresponding matching date for the reference group, and it ended with a diagnosis of EoE, death, emigration, or December 31, 2017, whichever occurred first. In reference individuals, follow-up also ended with a CeD diagnosis. Patients with a history of EoE before CeD were excluded from the analyses of future EoE risk.

We performed analyses stratified by sex (female vs male) and age at first CeD diagnosis (childhood ≤17 years; adulthood ≥18 years). Kaplan-Meier failure curves were plotted to show the probability of developing EoE. In a separate analysis, we compared the risk of EoE among patients with CeD to their unaffected siblings. Siblings were not matched, and so we adjusted for age and sex in sibling analyses.

HRs with 95% CIs that did not include 1.0 were considered statistically significant. All analyses accounted for the matching variables. Sensitivity analyses were also adjusted for education, country of birth, and other autoimmune diseases, with patients defined as exposed from their first non-CeD/non-EoE autoimmune diagnosis. In sibling comparisons, the data were stratified by family and adjusted for the same variables as in main analyses.

The R statistical software (version 4.0.5; R Foundation for Statistical Computing, Vienna, Austria) and the survival package (version 3.2)^18^ were used in statistical analyses.

### Ethical approval

The study was approved by the Stockholm Ethics Review Board (approval nos. 2014/1287-31/4 and 2018/972-32). The board waived informed consent because the study was register-based.^19^

## Results

We identified all individuals with biopsy-confirmed CeD from 2002 to 2017 in the ESPRESSO cohort and matched them with up to 5 reference individuals from the general population (Table I). All study participants were EoE-naive; 32 (0.12%) patients with CeD and 1 reference individual already had EoE at baseline (date of CeD diagnosis and matching) and were excluded. A total of 27,338 patients with CeD and 134,987 reference individuals were, hence, included in the final analysis.

**Table I:** Patients with CeD who met inclusion/exclusion criteria

Exclusion criteria	Patients with CeD (N = 27,473)	Reference individuals (N = 141,603)
Emigration issue	10 (0.04)	412 (0.29)
Death issue	10 (0.04)	1,348 (0.95)
Birthday issue	0 (0.00)	0 (0.00)
EoE before CeD	32 (0.12)	1 (0.00)
No match	83 (0.30)	4,861 (3.43)
Eligible	27,338	134,987

### Background data

Baseline characteristics of the study population are provided in Table II. The median age at CeD diagnosis was 27 years (interquartile range, 11-53), whereas 38.2% of the patients were diagnosed in childhood (≤17 years). About 95% of the patients with CeD were from a Nordic country and 63.3% were female patients. The average follow-up time was 8.2 years, with 38.0% having a follow-up time of 10 years or more. Finally, the educational level was similar in patients with CeD and general population reference individuals, whereas other autoimmune comorbidities were more frequent in patients with CeD (8.5%) than in reference individuals (2.6%) (Table II).

**Table II:** Summary statistics for patients with CeD and reference individuals

Characteristics	Patients with CeD (N = 27,338)	Reference individuals (N = 134,987)
Sex, n (%)		
Male	10,043 (36.74)	49,416 (36.61)
Female	17,295 (63.26)	85,571 (63.39)
Age (y) at start follow-up		
Mean ± SD	32.48 ± 24.52	32.06 ± 24.31
Median (IQR)	27.00 (11.00-53.00)	27.00 (11.00-52.00)
Range, minimum-maximum	0-95	0-95
<18	10,433 (38.16)	52,103 (38.60)
18 < 40	6,698 (24.50)	33,323 (24.69)
40 < 60	4,864 (17.79)	24,018 (17.79)
60	5,343 (19.54)	25,543 (18.92)
Country of birth, n (%)		
Nordic	25,882 (94.67)	120,782 (89.48)
Other	1,456 (5.33)	14,203 (10.52)
NA	0 (0.00)	2 (0.00)
Education, n (%)		
Compulsory school, ≤9 y	4,735 (17.32)	23,126 (17.13)
Upper secondary school, 10-12 y	7,387 (27.02)	36,307 (26.90)
College or university, ≥13 y	5,578 (20.40)	27,467 (20.35)
NA	9,638 (35.25)	48,087 (35.62)
Start of follow-up, n (%)		
2002-2009	15,606 (57.09)	77,004 (57.05)
2010-2017	11,732 (42.91)	57,983 (42.95)
Years of follow-up		
Mean ± SD	8.19 ± 4.46	8.52 ± 4.18
Median (IQR)	8.14 (4.61-11.91)	8.38 (5.05-12.02)
Range, minimum-maximum	0.00-15.99	0.00-15.99
<1	1,569 (5.74)	1,691 (1.25)
1 to <5	6,007 (21.97)	31,674 (23.46)
5 to <10	9,384 (34.33)	48,852 (36.19)
≥10 y	10,378 (37.96)	52,770 (39.09)
Comorbidity at start of follow-up, n (%)		
Autoimmunity∗	2,327 (8.51)	3,493 (2.59)

*IQR*, Interquartile range; *NA*, information missing.

∗ Common autoimmune disorders in patients with CeD included type 1 diabetes (occurring in 1036 individuals, equal to 3.80%), psoriasis (n = 368; 1.35%), thyroiditis/hyperthyroidism (n = 447; 1.60%), rheumatoid arthritis (n = 276; 1.01%), and SLE (n = 58; 0.21%).

### Risk of EoE in patients with biopsy-verified CeD

**Fig 1:**
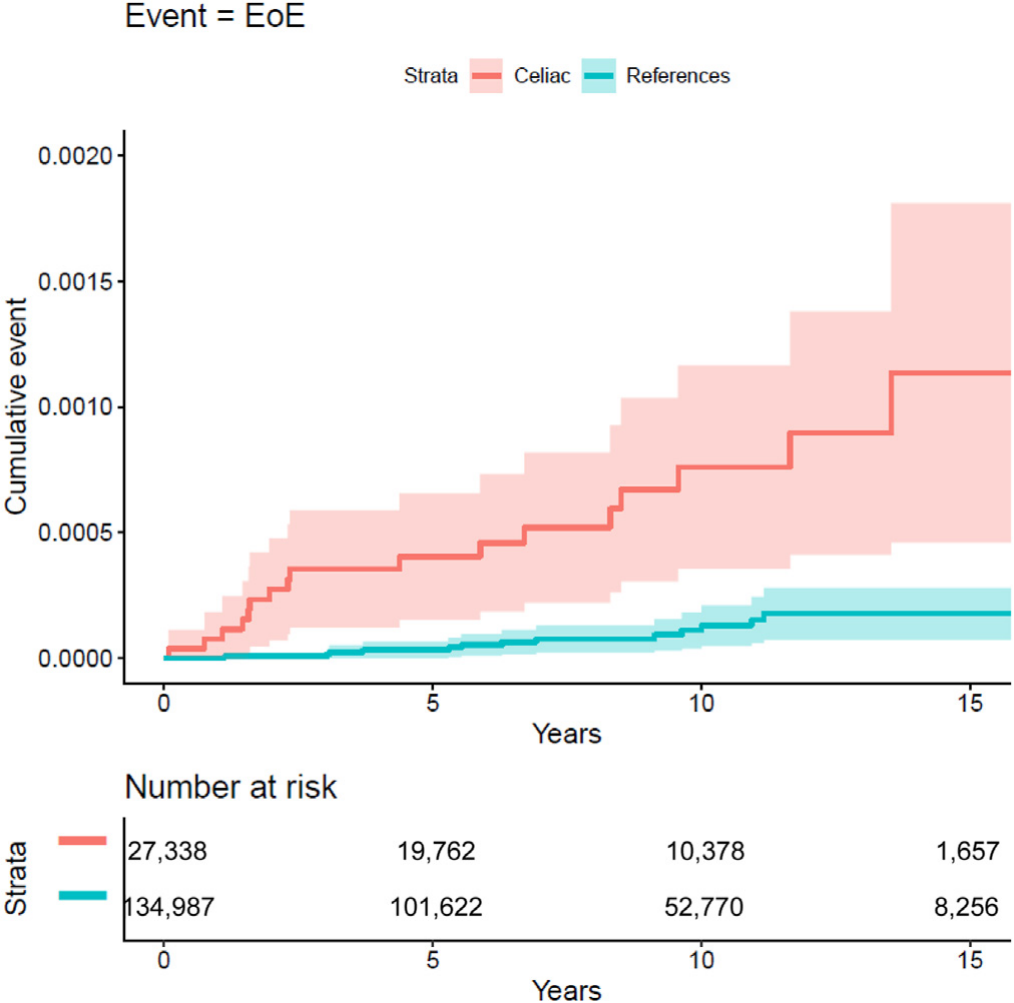
Plot of the cumulative incidence (event probability) over time for EoE in patients with CeD.

Seventeen patients with CeD (IR = 0.08/1000 person-years) and 13 reference individuals from the general population (IR = 0.01/1000 person-years) developed EoE during follow-up. This was equivalent to a 6.71-fold increased risk of later EoE in patients with CeD (95% CI, 3.26-13.81) (Table III; Fig 1).

**Table III:** Main analysis of EoE IRs and aHRs for patients with CeD and reference individuals

	Reference individuals (N = 134,987)	Patients with CeD (N = 27,338)
EoE events, N	13	17
IR (95% CI)	0.01 (0.01-0.02)	0.08 (0.04-0.12)
IRD (95% CI)	Reference	0.06 (0.03-0.1)
uHR	Reference	6.71 (3.26-13.81)
aHR	Reference	6.65 (3.19-13.85)

*aHR*, HR adjusted for age, sex, calendar year, county, education, and concomitant autoimmune disease; *IRD,* incidence rate difference; *uHR*, unadjusted HR.

Adjusting for education, country of birth, and CeD-related autoimmunity had only a marginal impact on the risk estimate with an adjusted HR of 6.65 (95% CI, 3.19-13.85) (Table III).

The increased risk of EoE was independent of sex (*P* heterogeneity = .08), and HRs were similar in childhood CeD and in adulthood (*P* heterogeneity = .23) (Table IV).

**TABLE IV:** aHRs for EoE in patients with CeD stratified by sex and age at start of follow-up

Characteristics	Reference individuals (EoE events/N)	Patients with CeD (EoE events/N); aHR (95% CI)	*P* heterogeneity
Sex			
Male	11/49,416	8/10,043; 4.06 (1.63-10.09)	
Female	2/85,571	9/17,295; 20.50 (4.32-97.30)	.0786
Age (y) at start of follow-up			
<18	7/52,103	13/10,433; 9.25 (3.61-23.72)	
≥18	6/82,884	4/16,905; 3.50 (0.99-12.42)	.2276

*aHR*, HR adjusted for age, sex, calendar year, county, education, and concomitant autoimmune disease.

### Sibling comparisons

Unaffected full siblings (n = 32,245) of 19,284 individuals with CeD were identified through the TPR and the Multigeneration Register. Twelve patients with CeD and 13 siblings developed EoE during follow-up. This was equal to IRs of 0.05 versus 0.07 per 1000 person-years. Hence, compared with their unaffected siblings, patients with CeD were at no greater risk of EoE, with an adjusted HR of 1.39 (95% CI, 0.55-3.51; Table V).

**Table V:** EoE IRs and aHRs for patients with CeD and their unaffected siblings

	Reference individuals (N = 32,245)	Patients with CeD (N = 19,284)
EoE events, N	13	12
IR (95% CI)	0.05 (0.02 to 0.08)	0.07 (0.04 to 0.13)
IRD (95% CI)	Reference	0.03 (−0.02 to 0.08)
uHR	Reference	1.28 (0.57 to 2.86)
aHR	Reference	1.39 (0.55 to 3.51)

*aHR*, HR adjusted for age, sex, calendar year, county, education, and concomitant autoimmune disease; *IRD,* incidence rate difference; *uHR*, unadjusted HR.

## Discussion

This nationwide cohort study of 27,338 individuals with biopsy-verified CeD from 2002 to 2017 found a more than 6-fold increased risk of EoE but low absolute risks. Compared with unaffected siblings, individuals with CeD were at no increased risk of EoE.

### Main findings in comparison with earlier studies

Several studies have previously reported a positive association between CeD and EoE.^9^, ^10^, ^11^^,^^20^, ^21^, ^22^, ^23^, ^24^ Thompson et al^21^ found an increased incidence of EoE among 1439 pediatric and adult cases of CeD compared with the general population, with an overall age- and sex-adjusted standardized incidence ratio of 16.0 (95% CI, 8.7-25.5). A 2008 case series reported a 3.2% prevalence of EoE among 221 children with CeD and encouraged physicians to perform routine esophageal biopsies when investigating for CeD.^10^ In 2015, Dharmaraj et al^11^ reported that 6 of 56 children with CeD developed EoE during a few years of follow-up (likely representing a risk increase although comparators were unavailable). A cross-sectional study in a US national pathology database also found an increased risk of EoE (+26%) in patients with CeD compared with subjects without CeD.^24^ Australian researchers have reported a 4% prevalence of EoE in 250 children with CeD.^20^ On the basis of these findings, several studies suggest that EoE should be ruled out in children undergoing endoscopy for suspected CeD^11^^,^^20^ or that esophageal biopsies be obtained regardless of esophageal endoscopy findings.^20^

In contrast, other articles report no association between CeD and EoE.^12^^,^^25^, ^26^, ^27^ A 2015 US study found that esophageal eosinophilia was not more common among 62 children with CeD compared with 91 children undergoing upper endoscopy for other reasons. The authors proposed an incidental and not causal association between the 2 entities.^25^ Similarly, Cristofori et al^27^ concluded that gastroenterologists should proceed to esophageal biopsy only in the presence of clinical symptoms of CeD to avoid unnecessary costs and difficulties in interpreting the results. A 2017 retrospective study of 10,201 children who underwent endoscopy reported a *decrease* in EoE among patients with CeD with an odds ratio of 0.29 (95% CI, 0.15-0.55).^26^ That article also presented a meta-analysis of 5 studies showing an overall odds ratio of 0.53 (95% CI, 0.36-0.80).^26^ Earlier in 2014, a systematic review by Lucendo et al^12^ included 30 publications and found that the prevalence of EoE in CeD ranged from 0% to 10.7%, whereas the prevalence of CeD in EoE varied between 0.16% and 57.1%. Clinical and methodological heterogeneity and lack of validated studies did not allow for quantitative summary calculations regarding prevalence data. Thus, the study by Lucendo et al did not rule out a true association between CeD and EoE. The authors concluded that available evidence did not support a positive association, emphasizing that the only epidemiological study with sufficient validity at the time argued against an association between the 2 disorders. However, EoE was rarely diagnosed before the 2000s and therefore updated analyses are needed.

Our cohort consisted of patients with CeD without a previous diagnosis of EoE, and, for this reason, we excluded individuals with a history of EoE before CeD from the analyses of future EoE risk. Although 32 patients with biopsy-verified CeD in the ESPRESSO cohort already had EoE at baseline, we found only 1 case of EoE before CeD among general population comparators. The small number of exposed comparators did not allow for further regression analyses but suggests that there is an association between EoE and CeD also before CeD diagnosis (ie, a bidirectional association).

CeD is significantly associated with other autoimmune disorders such as type 1 diabetes, Hashimoto thyroiditis, autoimmune hepatitis, and primary biliary cholangitis,^28^ whereas a 2022 study conducted by our research group found a bidirectional association between CeD and inflammatory bowel disease (both Crohn disease and ulcerative colitis).^29^ Similarly, there seems to be an elevated risk of multiple autoimmune disorders among individuals with EoE.^30^^,^^31^ Apart from a positive association with CeD, a 2016 US study found associations of EoE with Crohn disease, ulcerative colitis, and Hashimoto thyroiditis.^31^ Our research group, however, recently reported a positive association of EoE with only Crohn disease.^32^ In this study, we sought to take autoimmunity into account by adjusting for some potential confounding autoimmune disorders in our models. Yet, this adjustment did not change the results significantly (Table II).

Although EoE and CeD have distinct immunologic mechanisms, a T_H_2 inflammation for EoE,^33^ and, as proposed by some studies, a T_H_1 immune response to dietary gluten in the small intestinal mucosa for CeD,^34^, ^35^, ^36^ an impaired epithelial barrier also seems to play a role in the pathophysiology of both EoE^6^^,^^37^ and CeD^38^ as well as many other chronic noncommunicable diseases.^39^ According to the *epithelial barrier hypothesis*, the increase in barrier-damaging agents and substances because of industrialization and urbanization lies beneath the rise in both allergic and autoimmune conditions, and immune responses to dysbiotic microbiota crossing the damaged gut barrier may also be involved in the pathogenesis.^39^ Specifically, immune dysregulation in EoE seems to occur when environmental factors, such as food triggers and microbial imbalance, interact with the esophageal epithelium to elicit production of proinflammatory cytokines. Activated T_H_2 and regulatory T cells secrete bioactive cytokines, including IL-5, IL-13, and IL-4, leading to barrier disruption, tissue remodeling, eosinophilic inflammation, and progressive esophageal dysfunction.^40^ Arising from the interplay of genetic, environmental, and immunologic factors, CeD pathogenesis involves a complex cytokine network induced by gluten intake, including INF-γ produced by T_H_1 cells, IL-15, INF-α, and possibly IL-18^34^ and IL-21,^41^ whereas an increase in gut intraepithelial γδ T cells is considered a hallmark of CeD and their role in the diagnostic approach seems promising.^42^

A confounding issue is that both EoE and CeD are diagnosed by endoscopic biopsy, and it is common among both pediatric and adult gastroenterologists to biopsy all visualized areas of the upper GI tract during endoscopy. This has been advocated to increase case finding for CeD.^43^ Thus, having one of these conditions may predispose to the diagnosis of the other simply because both conditions can lead to multiple endoscopies. Either condition may in fact be asymptomatic.

Finally, absolute risks of EoE are dependent on the underlying incidence and prevalence of EoE. The prevalence of diagnosed EoE differs between countries, with higher rates in Western than in Asian populations.^44^ Dellon et al^5^ reported a period prevalence of 56.7 per 100,000 individuals but that US study was limited to a claims database, and it is possible that both comorbidity patterns and diagnostic workup differ between people with and those without health insurance. Data from our group found a prevalence of 10 to 15 per 100,000 individuals, but the life-time risk of biopsy-verified EoE was substantially higher (338 per 100,000 men and 123 per 100,000 women).^7^ Hence, we urge caution when extrapolating absolute risks in our study to countries with different health care systems.

### Strengths and limitations

The large sample size of 27,338 patients with CeD is among this study’s strengths. Consequently, we had substantial statistical power, something that allowed us to provide risk estimates also for different patient groups. Noteworthy, we found no difference in the HRs for EoE between female and male patients or in patients with childhood-onset CeD compared with those diagnosed in adulthood. To control for education and concomitant autoimmune conditions, we adjusted for these potential confounders in sensitivity analyses. In addition, we calculated absolute risks that are valuable from the perspective of communicating risks with patients and their families. Also, through the TPR, we retrieved data on emigration and mortality to calculate precise follow-up times. Loss to follow-up or bias due to socioeconomic status should not be an issue in this study, given that the Swedish population and health care registers have practically complete coverage and that the tax-funded public health care system^45^ allows equal access to all citizens independent of income or education.

The use of sibling comparisons is a way to minimize the influence of intrafamilial confounding. Our sibling analyses are, therefore, another strength of this study, allowing adjustment for the potential confounding effect of unmeasured shared familial characteristics. Intriguingly, compared with their siblings, patients with CeD were at no elevated risk of future EoE. This finding argues against CeD causing EoE; rather, the association may be explained by shared genetic or environmental components or by a different health-seeking pattern or surveillance compared with that of the general population. To our knowledge, ours is the first study in the field that has examined the potential influence of intrafamilial confounding.

Admittingly, the number of EoE cases in this study was low and underascertainment might be a possible limitation, although it is unlikely that this was differential by CeD status, that is, a source of substantial bias. In addition, our definitions of both CeD and EoE have high PPVs (95% and 89%, respectively).^17^^,^^46^ Particularly, we used histopathology data to identify individuals with these conditions. Also, our case ascertainment was nationwide, contrasting with earlier single-center studies that may have oversampled cases with severe disease or comorbidity. The population-based design should have minimized selection bias. However, our results might mostly apply to Western populations, considering that most individuals in our cohort originated from the Nordic countries.

Limitations of this study also include lack of data on gluten consumption. Hence, we could not examine the potential role of dietary adherence in CeD on the risk of EoE. Also, we did not have data on symptoms that might have prompted EoE investigations for CeD or EoE, and we cannot rule out that the association with EoE might apply especially to more severe CeD. According to 2012 European guidelines, a nonbiopsy CeD diagnosis is possible in a subset of children with high antibody titers, typical symptoms, and genetic predisposition.^47^ We may, thus, have missed some patients with childhood-onset CeD diagnosed after 2012. Another drawback is the lack of data regarding other GI disorders including gastroesophageal reflux disease coexisting at the time of CeD diagnosis, which might have affected the risk of EoE. Finally, considering that even today one-third of patients with EoE have a persistently long diagnostic delay of 10 years or more from onset of symptoms to diagnosis,^48^ the mean follow-up time of 8.2 years in our study might be another potential limitation. Finally, Swedish registers do not contain data on race, and hence we cannot rule out that our findings mainly apply to Western populations considering that most individuals in our cohort originated from the Nordic countries.

This nationwide population-based cohort study found an almost 7-fold increased risk of new-onset EoE in individuals with CeD. The absolute risks, though, were low, and the positive association almost disappeared when we compared with siblings, implying that the association might be explained by shared intrafamilial factors or altered health-seeking patterns following CeD diagnosis. We encourage physicians to consider coexistent EoE in patients with CeD with persistent esophageal symptoms.

## Disclosure statement

This study was supported by grants from Örebro County Council (avtal om läkarutbildning och forskning [ALF]). A.U. is supported by the Consortium of Eosinophilic Gastrointestinal Disease Researchers.

Disclosure of potential conflict of interest: A. Uchida consults for Sanofi-Regeneron and AstraZeneca. J. F. Ludvigsson coordinates a study on behalf of the Swedish Inflammatory Bowel Disease Register (SWIBREG), which has received funding from Janssen Corporation; has also received financial support from Merck Sharp and Dohme (MSD) developing an article reviewing national health care registers in China; and is currently discussing potential research collaboration with Takeda. The rest of the authors declare that they have no relevant conflicts of interest.
